# 970. Prevalence of Heterotopic Ossification May Be Underreported in the Literature

**DOI:** 10.1093/jbcr/irag033.567

**Published:** 2026-04-06

**Authors:** Andria Martinez, Renée Warthman, Suzanne C Osborn, Philomene Spadafore, Jonathan Subramaniam, Natalie V Kesler, Stephanie Strong, Rosalyn A Katz, Erica Whetten, Karen J Richey, Kevin N Foster

**Affiliations:** Diane & Bruce Halle Arizona Burn Center - Valleywise Health, Phoenix, AZ; Diane & Bruce Halle Arizona Burn Center - Valleywise Health, Phoenix, AZ; Diane & Bruce Halle Arizona Burn Center - Valleywise Health, Phoenix, AZ; Diane & Bruce Halle Arizona Burn Center - Valleywise Health, Phoenix, AZ; Diane & Bruce Halle Arizona Burn Center - Valleywise Health, Phoenix, AZ; Diane & Bruce Halle Arizona Burn Center - Valleywise Health, Phoenix, AZ; Diane & Bruce Halle Arizona Burn Center - Valleywise Health, Phoenix, AZ; University of Wisconsin-Madison, Madison, WI; Valleywise Health Medical Center, Phoenix, AZ; Diane & Bruce Halle Arizona Burn Center - Valleywise Health, Phoenix, AZ; Diane & Bruce Halle Arizona Burn Center - Valleywise Health, Phoenix, AZ

## Abstract

**Introduction:**

Heterotopic ossification (HO) is a potential burn complication that can significantly impact function, something these authors feel remains underreported in the literature, and physical assessment of HO is a delicate and perishable skill. Previous studies examining the prevalence of HO place the incidence rate at approximately 4%. Early detection of HO may improve patient outcomes, however early clinical assessment and management have yet to be clearly defined. A previous study identified that the presence of three clinical symptoms; decreased range of motion (ROM), disproportionate pain to affected joint, and change in end feel correlated with burn therapists’ ability to detect HO formation to the elbow prior to radiologic detection. Our research aims to demonstrate that therapy driven methods of HO assessment are more sensitive to the prevalence of this complication. The purpose of this study was to explore incidence of HO in a large sample to test our hypothesis, which would have the associated benefit of reinforcing or refuting data from prior studies on causality of HO.

**Methods:**

We performed a 10-year retrospective study of adult patients who suffered ≥20% total body surface area (TBSA) burns at a single burn center. Subjects were grouped into 2 cohorts, those who developed HO (HO) at any joint and those who did not develop HO (No-HO). Collected variables included demographics, burn characteristics (TBSA, depth, location), escharotomy, graft location, imaging, therapy assessments, clinical symptoms (disproportionate pain with limited range of motion (ROM), and change in end feel), and hospital data. Descriptive statistics, group comparisons, and multivariable logistic regression were conducted using Python 3.11.

**Results:**

Of a total of 339 patients met inclusion criteria, 52 (15.3%) developed HO and 287 did not. Compared with No-HO, the HO group had larger mean TBSA injuries 43.2% vs 29.5% (*p*<.001) and longer mean length of stay 69.5 vs 27.0 days (*p*<.001). Age did not differ significantly, with HO at 39 vs No-HO 35 years. In exploratory analyses HO was more frequently observed among joints with ≥1 recorded symptom. Upper extremity grafting was independently associated with HO (adjusted OR 3.83, 95% CI 1.49-9.80, *p*=.021).

**Conclusions:**

Incidence of HO in our sample set is higher than what was reported in the literature, supporting our contention that HO may be underreported. Larger TBSA, longer LOS, and upper extremity grafting were corroborated as independent risk factors for developing HO in our sample data. This study also suggests that patients who develop HO may favor higher injury severity, consistent with previously published research.

**Applicability of Research to Practice:**

This study may help further inform clinical decision making regarding possible development of HO. More consistent attention to the vexing problem of HO should be made in the literature and research.

**Funding for the study:**

N/A.

